# Skin dysbiosis and *Cutibacterium acnes* biofilm in inflammatory acne lesions of adolescents

**DOI:** 10.1038/s41598-022-25436-3

**Published:** 2022-12-06

**Authors:** Ilaria Cavallo, Francesca Sivori, Mauro Truglio, Flavio De Maio, Federica Lucantoni, Giorgia Cardinali, Martina Pontone, Thierry Bernardi, Maurizio Sanguinetti, Bruno Capitanio, Antonio Cristaudo, Fiorentina Ascenzioni, Aldo Morrone, Fulvia Pimpinelli, Enea Gino Di Domenico

**Affiliations:** 1 Microbiology and Virology, IRCCS San Gallicano Institute, Rome, Italy; 2 Cutaneous Physiopathology, San Gallicano Dermatological Institute, IRCCS, Rome, Italy; 3 Dipartimento di Scienze di Laboratorio e Infettivologiche, Fondazione Policlinico Universitario “A. Gemelli” IRCSS, Rome, Italy; 4 Department of Biology and Biotechnology Charles Darwin, Sapienza University of Rome, Rome, Italy; 5 Biofilm Pharma SAS, Saint-Beauzire, France; 6 Dipartimento di Scienze Biotecnologiche di Base, Cliniche Intensivologiche e Perioperatorie - Sezione di Microbiologia, Università Cattolica del Sacro Cuore, Rome, Italy; 7 Clinical Dermatology, San Gallicano Dermatological Institute, IRCCS, Rome, Italy; 8 Scientific Direction, IRCCS San Gallicano Institute, Rome, Italy

**Keywords:** Microbiology, Antimicrobials, Biofilms, Clinical microbiology, Microbial communities

## Abstract

Acne vulgaris is a common inflammatory disorder affecting more than 80% of young adolescents. *Cutibacterium acnes* plays a role in the pathogenesis of acne lesions, although the mechanisms are poorly understood. The study aimed to explore the microbiome at different skin sites in adolescent acne and the role of biofilm production in promoting the growth and persistence of *C. acnes* isolates. Microbiota analysis showed a significantly lower alpha diversity in inflammatory lesions (LA) than in non-inflammatory (NI) lesions of acne patients and healthy subjects (HS). Differences at the species level were driven by the overabundance of *C. acnes* on LA than NI and HS. The phylotype IA1 was more represented in the skin of acne patients than in HS. Genes involved in lipids transport and metabolism, as well as potential virulence factors associated with host-tissue colonization, were detected in all IA1 strains independently from the site of isolation. Additionally, the IA1 isolates were more efficient in early adhesion and biomass production than other phylotypes showing a significant increase in antibiotic tolerance. Overall, our data indicate that the site-specific dysbiosis in LA and colonization by virulent and highly tolerant *C. acnes* phylotypes may contribute to acne development in a part of the population, despite the universal carriage of the microorganism. Moreover, new antimicrobial agents, specifically targeting biofilm-forming *C. acnes*, may represent potential treatments to modulate the skin microbiota in acne.

## Introduction

Acne vulgaris is a chronic inflammatory skin disorder of the pilosebaceous unit, affecting 67–95% of adolescents worldwide^1–3^. In adults, its prevalence and incidence have grown, especially among women^4–6^. The psychological impact of acne is substantial, causing a detrimental impact on life quality^7,8^. Acne is characterized by increased sebum production, leading to non-inflammatory (NI) comedones and inflammatory lesions (LA) such as papules, pustules, or nodules, mainly on the face and on the back and chest^9^. The etiology is associated with changes in sebum production under androgen control, an altered keratinization process, and an increased release of inflammatory mediators^10–13^. Dysbiosis and follicular colonization by *Cutibacterium acnes* have been linked to the pathophysiology of inflammatory acne^14,15^. However, the role of *C. acne* remains unclear due to its ubiquitous distribution both in the sebaceous areas of healthy skin and inflammatory acne lesions and its highly variable density in the skin of different individuals^15^. Although generally regarded as a low-virulence bacterium and a human skin commensal, *C. acnes* can be considered an opportunistic pathogen associated with invasive skin and soft tissue infections and implant-associated infections^16–18^. Indeed, C*. acnes*, by producing multiple virulence factors such as lipases, proteases, and the Christine Atkins Munch-Petersen (CAMP) factors, can trigger inflammation and host tissue damage^19–24^. Besides, *C. acnes* proliferation in the pilosebaceous unit can induce the upregulation of different proinflammatory cytokines by keratinocytes, sebocytes, or peripheral blood mononuclear cells^25–28^. *C. acnes* strains are categorized into the six phylotypes IA1, IA2, IB, IC, II, and III, correlated with a variable body distribution, clinical conditions, antimicrobial susceptibility profile, and inflammatory properties^29–32^. *C. acnes* IA1 is the predominant phylotype isolated from moderate to severe acne. In contrast, IB, II, and III phylotypes are more commonly isolated from soft tissues and medical implant infections^33–35^. Notably, IA1 isolates exhibit a more virulent profile, including a greater production of β-defensin 2 from cultured keratinocytes and higher lipase activity levels than other phylotypes associated with healthy skin^36,37^. These observations suggest that the apparent loss of phylotype diversity in acne patients and the dominance of IA1 virulent strains may reflect a dysbiotic shift within a follicle linked to microenvironmental changes^38^. In particular, biofilm production has been linked to specific *C. acnes* phylotypes, thus suggesting a possible association with the chronic colonization of the pilosebaceous unit observed in acne patients^39–42^. Previous works have demonstrated that *C. acnes* form biofilms in the follicles of acne patients^43^, suggesting that this condition may lead to a homeostatic imbalance of the microbiota^15^. Indeed, the chronic bacterial persistence and relapse following antibiotic therapy are strongly indicative of biofilm-related colonization^44^. Although biofilm is considered a primary factor that ensures *C. acnes* persistence during acne antibiotic treatment ^45^, factors that promote early bacteria adhesion and biofilm formation have not been identified yet^46^. This study analyzed the process of *C. acnes* colonization and its relation to the skin microbiota in patients with severe acne from NI and LA and healthy subjects (HS). Besides, using a metagenomic approach, we characterized the genetic background and the phylotype-dependent biofilm production of *C. acnes* isolates with regard to the dynamics of early adherence, the overall amount of biofilm biomass, and morphology. Finally, we also investigated the contribution of biofilm to antibiotic tolerance of *C. acnes* isolates.

## Results

### Demographic and baseline data

A total of ten patients affected by severe acne vulgaris, and ten healthy control subjects (HS), with no history of acne were included in the study. The two groups were matched for age and sex. The demographic and clinical characteristics are summarized in Table 1.

**Table 1:** Demographic and clinical characteristics of acne patients at inclusion.

Baseline demographic and clinical characteristics of acne patients	N = 10
Sex, Female/Male	6/4
Age (years), Mean ± SD; Median [Min–Max]	14.1 ± 2; 14 [11–18]
Dermatological examination	No signs apart from acne
Facial acne severity with the GEA scale Grade 4–5 (Severe)	10

### Skin surface microbiota of healthy subjects and acne patients

Analysis of 2,275,010 reads (min. number of reads: 43,111; max. number of reads: 187,496) grouped into 2037 taxa. Specifically, 2,139,830 reads grouped into 916 taxa were collected after filtering out singletons and taxa with a prevalence lower than 5%. After rarefaction at a sample depth of 40,000 reads, 880 taxa were recovered, and alpha and beta diversity were evaluated. Two samples were excluded from the 10 healthy controls due to low quality and library size.

**Figure 1:**
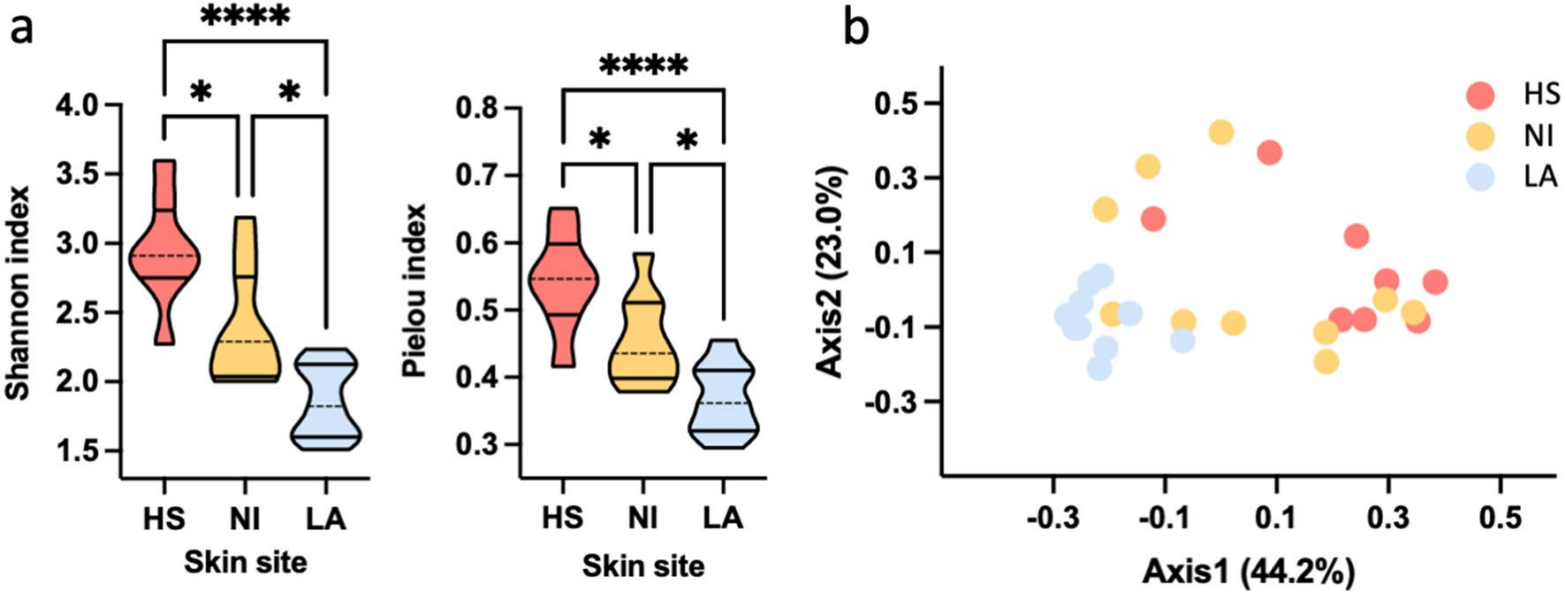
Loss of diversity characterizes inflammatory acne. Skin microbiota was evaluated on samples collected from the skin of ten healthy subjects (HS) and non-inflammatory (NI), and inflammatory lesions (LA) of ten acne patients. (**a**) Alpha diversity was calculated using the Shannon diversity index (HS vs. NI, *P* = 0.0209; HS vs. LA, *P* < 0.0001; LA vs. NI, *P* = 0.0118) and Pielou Evenness index (HS vs. NI, *P* = 0.0241; HS vs. LA, *P* < 0.0001; LA vs. NI, *P* = 0.0136). Statistical differences were determined using the Kruskal–Wallis test. (**b**) Bray Curtis beta diversity was calculated at the genus level and represented as principal coordinate analysis (PCoA). PERMANOVA test was used to assess significance. *, *P* < 0.05; **, *P* < 0.01; ***, *P* < 0.001, ****, *P* < 0.0001.

Alpha diversity of the skin of ten healthy subjects (HS) and non-inflammatory (NI) and inflammatory lesions (LA) of ten acne patients was evaluated using the Shannon diversity index and Pielou evenness index. Interestingly, a significant (*P* = 0.0002) decrease in Shannon index was observed in both NI (Mean ± SD = 2.39 ± 0.41) and LA (Mean ± SD = 1.86 ± 0.26) compared to HS (Mean ± SD = 2.95 ± 0.39), (Fig. 1a). Similarly, a significant (*P* = 0.0004) decrease in Pielou index was reported in NI (Mean ± SD = 0.45 ± 0.07) and LA (Mean ± SD, 0.37 ± 0.05) samples than in HS (Mean ± SD, 0.54 ± 0.07), (Fig. 1a). Bray Curtis beta diversity, represented as principal coordinate analysis (PCoA), corroborated previous findings showing a distinctive spatial clusterization of HS samples and subjects with LA (PERMANOVA test: *P* = 0.001, R^2^ = 0.3522) (Fig. 1b).

**Figure 2:**
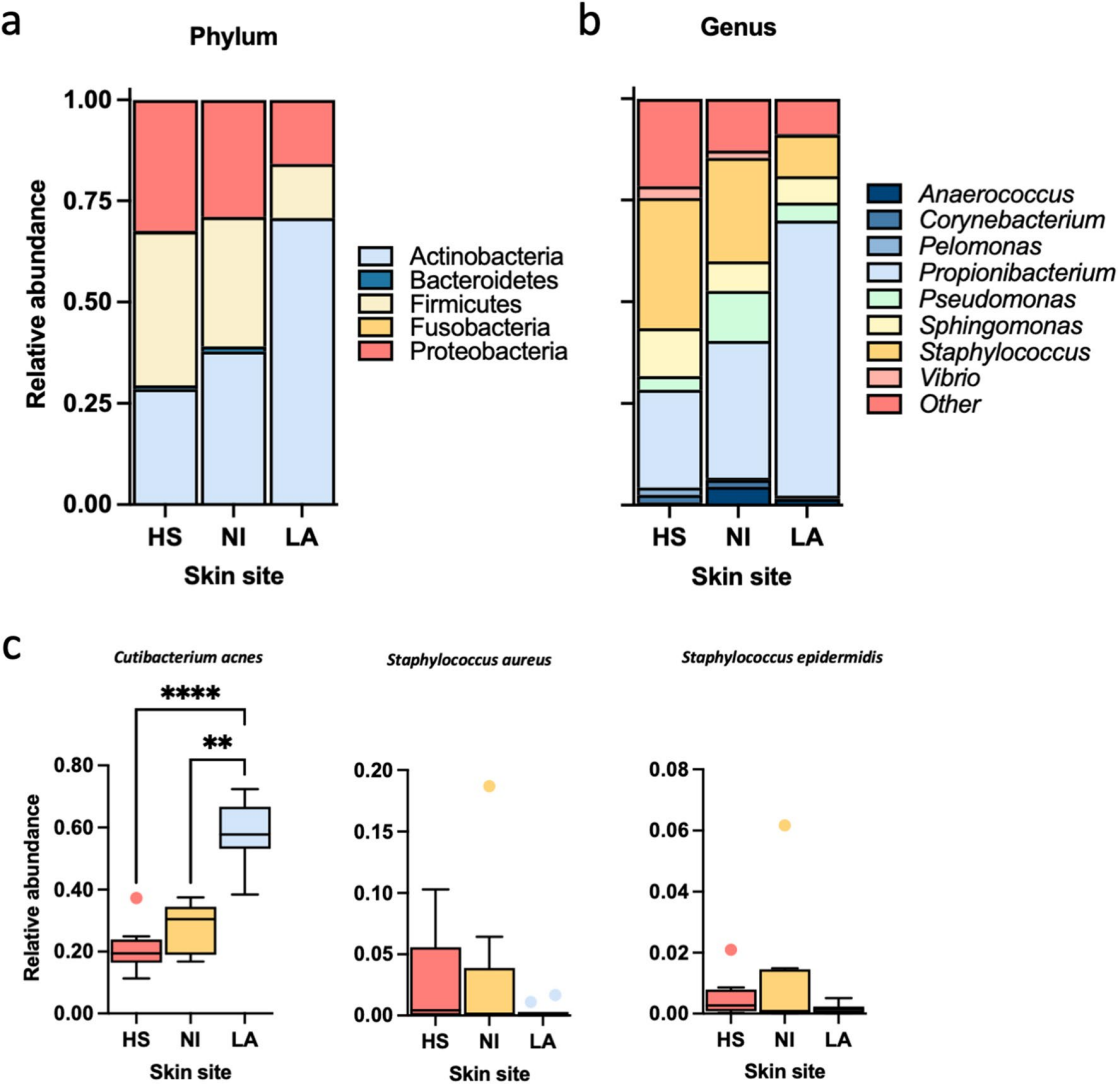
Microbiota variation between healthy subjects and acne patients. Skin microbiota was evaluated on samples collected from the skin of ten healthy subjects (HS) and non-inflammatory (NI) and inflammatory lesions (AL) of 10 acne patients. Relative abundances at the phylum level (**a**) and top eight genera (**b**) were represented in a stacked bar plot. (**c**). Relative abundance was evaluated at the species level for *Cutibacterium acnes*, *Staphylococcus aureus,* and *Staphylococcus epidermidis* relative abundances. Significance was assessed by using the Kruskal Wallis static test. *, *P* < 0.05; **, *P* < 0.01; ***, *P* < 0.001, ****, *P* < 0.0001.

Important site-dependent differences were identified in bacterial abundance at phylum and genus level (Fig. 2). The relative abundance of *Actinobacteria* increased in LA specimens compared to what was observed for *Firmicutes* and *Proteobacteria* (Fig. 2a). Accordingly, the relative abundance of *Actinobacteria* accounted for 30% of the microbial community in HS, 38% in NI, and increased to 71%in LA (*P* < 0.001). Differently, *Firmicutes* and *Proteobacteria* appeared more abundant in HS (38% and 32%) and NI (32% and 29%) compared to LA (13%, 5%).

A point worth noting is that NI appeared more variable than HS and LA specimens, especially regarding *Firmicutes* and *Proteobacteria* abundances. The inverse relationship observed for *Firmicutes* and *Actinobacteria* phyla was confirmed by analyzing the top ten genera (Fig. 2b). The most dramatic difference was the dominance of *Propionibacterium* and the reduction in the relative abundance of *Staphylococcus* genus in LA compared to HS and NI. At the species level (Fig. 2c), *Cutibacterium acnes* showed a significant difference between different sites (*P* < 0.0001). Conversely, the relative abundance of *Staphylococcus aureus* and *Staphylococcus epidermidis* did not change significantly in HS, NI, and LA.

### Typing and whole-genome sequencing of *C. acnes* isolates

To determine the characteristics of the culturable *C. acnes,* bacterial colonies were isolated from site-matched skin in healthy controls (N10) and patients with acne (N10). The WGS results of *C. acnes* isolates are reported in Table 2. The 20 strains were distributed among five clonal complexes (CCs), with the most frequent being CC1 (n = 9; 45.0%), followed by CC3 (n = 5; 25%) and CC4, CC5 and CC6 (n = 2; 10% each). The overall distribution of CCs showed a significant difference between HS and LA (*P* = 0.03). The most represented *C. acnes* phylotype was IA1 (n = 16; 80%) followed by type IB (n = 2; 10%) and II (n = 2; 10%) strains. The phylotype analysis revealed a significant difference in the distribution between HS and LA (*P* = 0.03), with the IA1 significantly more abundant in LA than in HS (*P* = 0.03).

**Table 2:** Multilocus sequence typing (MLST) and phylotype profiles of the *C. acnes* strains isolated from the skin of healthy subjects (HS) and the lesional area of acne patients (LA).

Source	Sequence typing	Clonal complex	Phylotype
HS	6	CC6	II
HS	4	CC4	IA1
HS	5	CC5	IB
HS	1	CC1	IA1
HS	3	CC3	IA1
HS	1	CC1	IA1
HS	1	CC1	IA1
HS	5	CC5	IB
HS	7	CC6	II
HS	4	CC4	IA1
LA	1	CC1	IA1
LA	3	CC3	IA1
LA	3	CC3	IA1
LA	115	CC3	IA1
LA	1	CC1	IA1
LA	3	CC3	IA1
LA	1	CC1	IA1
LA	1	CC1	IA1
LA	1	CC1	IA1
LA	1	CC1	IA1
ATCC 11827	1	CC1	IA1

**Figure 3:**
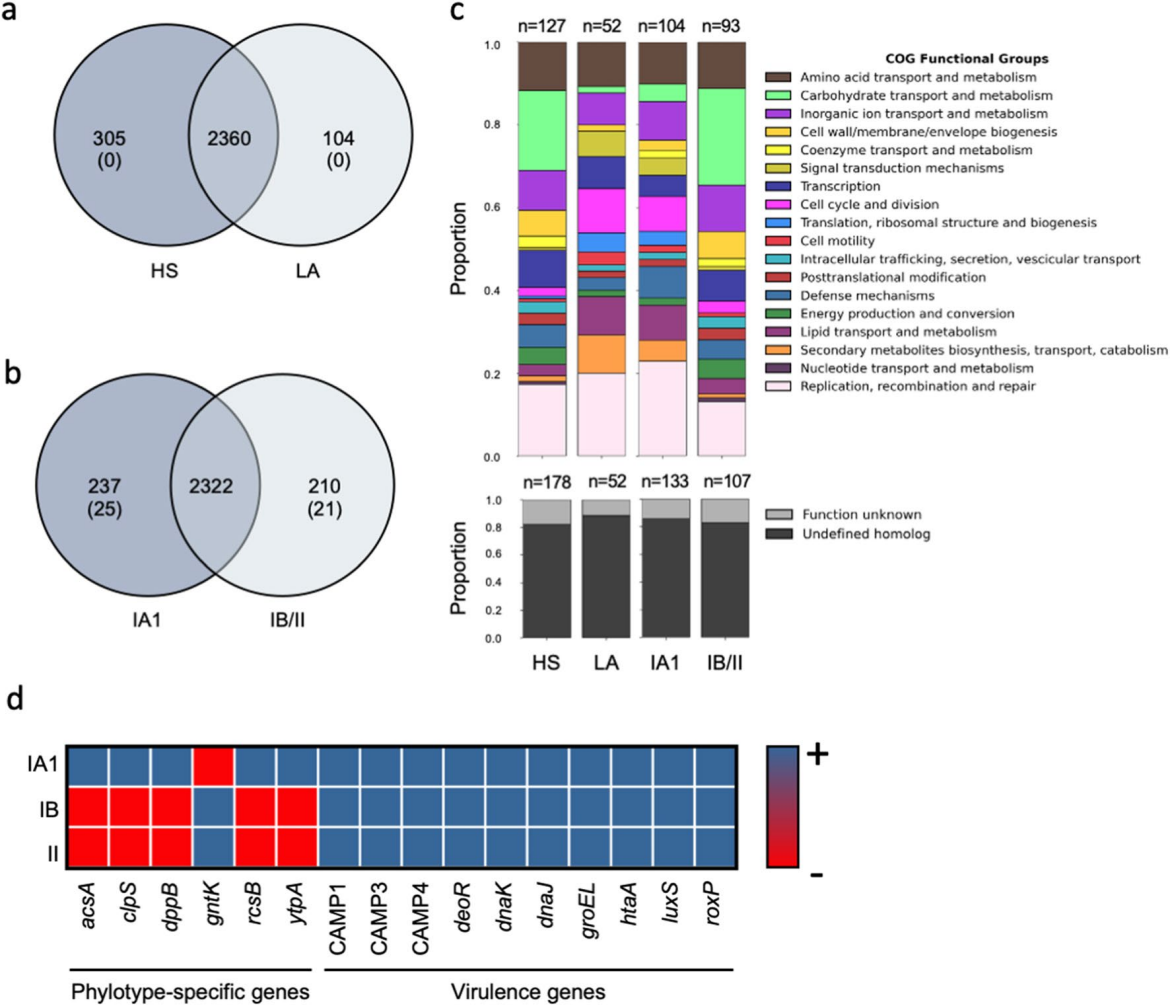
Shared and unique genes in 20 *C. acnes* strains. Venn diagrams display the distribution of shared and unique genes between (**a**) *C. acnes* strains from healthy subjects (HS) and the lesional area of acne patients (LA) and (**b**) phylotypes IA1 and IB/II. Overlapping regions show the genes conserved within strains. The number between brackets represents the unique genes present in all the strains of a particular group. (**c**) The stacked bar chart of Cluster of Orthologous Genes (COGs) functional category proportions is based on the unique genes in all groups. The n above each group indicates the absolute count of COGs identified in each group. (**d**) Similarity matrix categories represent the presence (blue; +) or the absence (red, -) of *C. acnes* virulence genes and phylotype-specific genes.

The number of putative protein-coding sequences in different genomes varied between 2332 and 2427, with an average of 2380. To assess genomic conservation across *C. acnes* isolates, the coding sequences were used to determine the pan-genome. This analysis revealed a total of 2769 genes representing the pan-genome of 20 *C. acnes* strains. To investigate the genetic determinants differentiating between different *C. acnes* isolates, unique genes were identified by querying the set for group-specific genes. Specifically, 305 genes occurred uniquely in *C. acnes* strains isolated from HS, whereas 104 unique genes were identified in *C. acnes* isolated from LA (Fig. 3a).

Nevertheless, no unique genes were present in all the strains isolated from different skin sites.

The unique and core genes within phylotypes were identified to investigate further the possible clustering in *C. acnes* isolates (Fig. 3b). The genetic analysis revealed that the IA1 phylotype contained a higher number of unique genes (2559) than the IB/II (2532). The unique gene analyses showed that 237 genes occurred uniquely in the IA1 phylotype while 210 characterized the IB/II group (Fig. 3b). Of the 237 unique genes, 25 were present in all the type IA1 strains and 21 in the IB/II strains, representing the key genetic content distinguishing type IA1 strains from the IB/II clades (Fig. 3b). Functional categories were analyzed and reported in Fig. 3c. Notably, of the 237 unique genes present in IA1, 43.9% were assigned to specific functions, while 56.1% remained with unknown functions. Similarly, in IB/II 44.3% of the 210 unique genes were assigned to particular functions, and 55.7% had unknown functions (Fig. 3c). Interestingly, for the phylotype IA1, five unique genes with known functions were identified (*acsA, clpS, dppB, rcsB, ytpA*). In particular, AcsA and RcsB are positively involved in biofilm formation in different bacterial species. ClpS is an essential gene product involved in a highly conserved mechanism that targets specific proteins for destruction in prokaryotes and eukaryotes; DppB is a dipeptide ABC transporter required for bacterial growth, and the lysophospholipase YtpA is a virulence factor in *C. acnes* contributing to host-tissue degradation and inflammation. The type IB/II clades only displayed one unique gene with known function shared across all strains, the Shikimate kinase *gntK*. Putative virulence factor genes, coding for the iron acquisition protein (HtaA), the heat shock proteins (DnaK, DnaJ, and GroEL), the *luxS* gene, which is involved in the synthesis of the autoinducer 2, the radical oxygenase RoxP and the Christie–Atkins–Munch–Petersen (CAMP) factors 1, CAMP 2, and CAMP 4 were ubiquitous in every *C. acnes* strain, and not associated with specific genotypes (Fig. 3d). The co-hemolytic activity of the CAMP factor was further confirmed on blood agar plates for all *C. acnes* isolates. To further decipher the relationship among the phylotype IA1 strains, we generated a phylogenomic tree based on the core genome variants alignment and a phylogenetic tree based on the core pangenome allelic variations (supplemental information). The trees showed the samples clustered by clonal complex and sequence typing but not by the skin site (HS vs. LA).

### *Cutibacterium acnes* adhesion and biofilm formation

**Figure 4:**
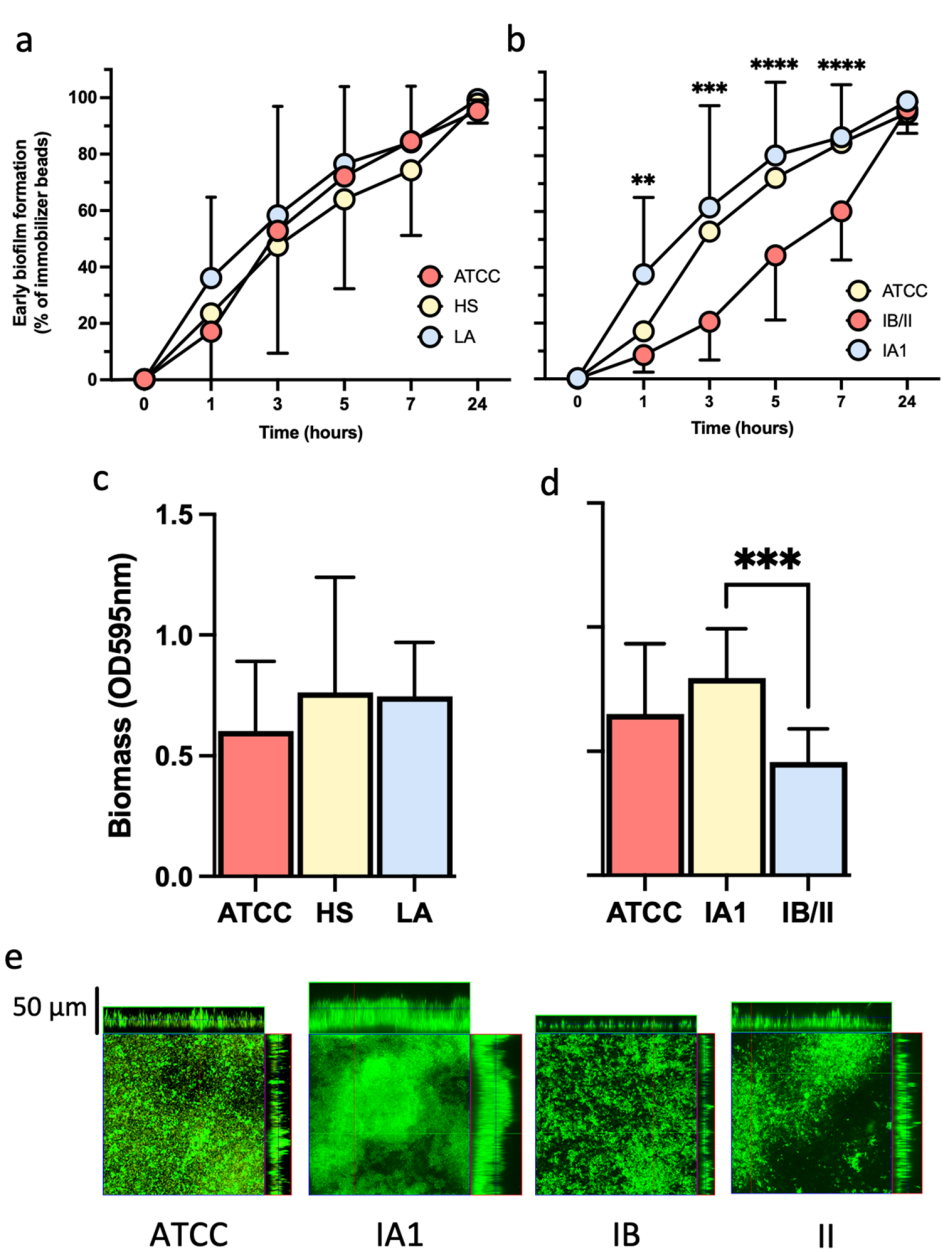
Biofilm formation of *C. acnes* strains. **a** Kinetic of bacterial adhesion was measured using the BioFilm Ring Test for *C. acnes* strains isolated from healthy subjects (HS) and the lesional skin of acne patients (LA) and (**b**) according to the phylotypes IA1, IB, II. *C. acnes* ATCC 1182 was used as a reference control strain. The mean and corresponding standard errors for three independent experiments of duplicate samples for each time point are shown. (**c**,**d**) The amount of biofilm biomass after 72 h was measured by the crystal violet assays (optical density (OD) at 595 nm). The mean and corresponding standard errors for three independent experiments of duplicate samples for each time point are shown. (**e**) Confocal microscopy image of biofilm formation of different *C. acnes* isolates and the *C. acnes* ATCC 1182 after 72 h of incubation in BHI at 37 °C. Significance was assessed by using the Kruskal Wallis static test. *, *P* < 0.05; **, *P* < 0.01; ***, *P* < 0.001, ****, *P* < 0.0001.

*Cutibacterium acnes* biofilms are frequently observed in patients with acne^47^. Moreover, the hierarchical clustering analysis indicated that the biofilm-related genes AcsA and RcsB were present in IA1 isolates but not in IB/II, suggesting possible differences in biofilm production. Therefore, the biofilm-forming ability of different *C. acnes* isolates was investigated. First, the early bacterial adhesion kinetics was measured at 0, 1, 3, 5, 7, and 24 h. No significant differences in the early biofilm formation of the *C. acnes* strains from HS and LA were observed during all the periods analyzed (Fig. 4a). Moreover, since IA1 colonized both HS and LA, the kinetics of early biofilm formation were analyzed on the phylotypes groups. In particular, the *C. acnes* strains belonging to the phylotypes IA1 were significantly faster in the early biofilm formation, than the phylotypes IB/II, after 1 (*P* = 0.0016), 3 (*P* = 0.0004), 5 (*P* < 0.0001) and 7 (*P* < 0.0001) hours. Nevertheless, after 24 h, all the strains analyzed achieved a comparable inhibition of the magnetic microparticles independently from the phylotype.

Biofilm formation was further quantified by determining the biomass with crystal violet (CV) 72 h after incubation (Fig. 4b). The strains isolated from LA produced a comparable biomass level to those from HS and *C. acnes* 11827 ATCC (Fig. 4c,d). Notably, IA1 isolates formed significantly (*P* = 0.0004) more biofilm than the IB/II strains, suggesting that the phylotype impacted biomass production rather than the isolation site (Fig. 4d).

The morphology of biofilms was also investigated using confocal microscopy (Fig. 4e) for the reference strains *C. acnes* 11827 ATCC and the phylotypes IA1, IB, and II. Most of the *C. acnes* isolates produced a thick biofilm after 72 h, as indicated by the presence of cell aggregates and pillars of more than 20 µm (z projection). Consistent with the results obtained by microtiter plate tests, the IA1 isolates exhibited increased biomass and thickness compared to the IB and II isolates.

**Figure 5:**
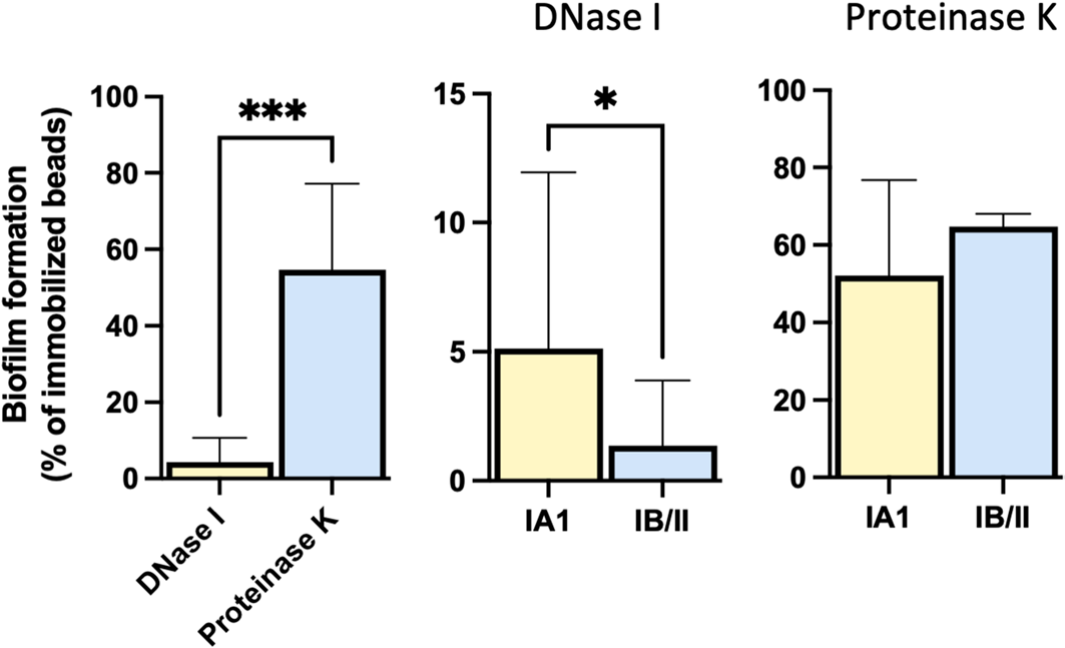
DNase I and Proteinase K reduce *C. acnes* adhesion. Comparison of extracellular DNA and proteins on early surface adhesion for the IA1 and IB/II phylotypes. Results are expressed as relative differences (Eq. 1) in the amounts of biofilm as measured by the BioFilm Ring Test after 6 h of incubation in the presence of DNase and Proteinase K compared with untreated control strains. Data represent means and the corresponding standard errors of two independent experiments analyzed in duplicate; *, *P* < 0.05; **, *P* < 0.01; ***, *P* < 0.001, ****, *P* < 0.0001 using the Mann–Whitney test.

The biofilm matrix of *C. acnes* is composed of carbohydrates, proteins, and eDNA^48^. The impact of DNase I and proteinase K on biofilms has been used to investigate the contribution of eDNA and proteins in early adhesion and biofilm formation^49^. The treatment with DNase I and proteinase K caused a substantial reduction in the initial attachment to abiotic surfaces of *C. acnes* strains of all phylotypes (Fig. 5). However, DNase I treatment showed a significant (*P* < 0.001) reduction compared with proteinase K in the initial attachment of *C. acnes* strains suggesting that eDNA is critical for the initial adhesion. In particular, the reduction in the initial attachment to abiotic surfaces of *C. acnes* is significantly more pronounced in the phylotypes IB/II compared to IA1 (Fig. 5).

### Assessment of the antimicrobial susceptibility profiles

**Figure 6:**
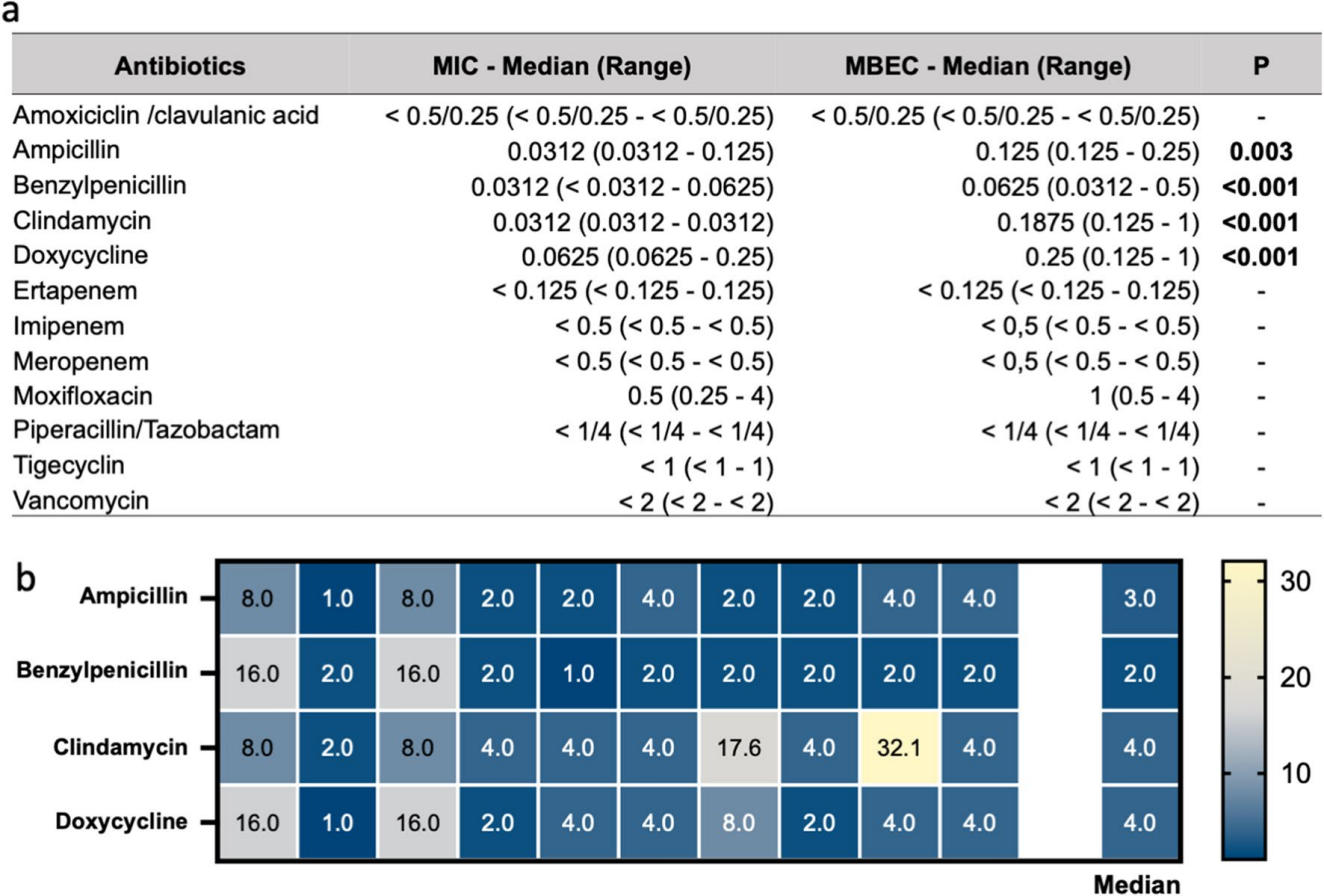
Antimicrobial tolerance in *C. acnes* isolates. (**a**) Antimicrobial susceptibility testing against ten *C. acnes* strains (phylotype IA1) isolated from the lesional skin of acne patients in planktonic and biofilm growth measured as minimum inhibitory concentration (MIC) and minimal biofilm eradication concentration (MBEC) for the indicated antibiotics. (**b**) Heat map showing the biofilm tolerance (BT), calculated as the ratio MBEC/MIC for ampicillin, benzylpenicillin, clindamycin, and doxycycline. Yellow indicates high BT values, and blue represents low BT for the indicated antibiotics. Statistical differences were determined using the Kruskal–Wallis test, followed by Dunn’s post hoc test for multiple comparisons.

The activity of different antibiotics by the minimum inhibitory concentration (MIC) and minimal biofilm eradication concentration (MBEC) was measured in planktonic and biofilm growth on the ten *C. acnes* phylotype IA1 strains isolated from LA. The MIC values for each antibiotic are summarized in Fig. 6. The susceptibility profiles apparently contradicted the lack of response to several anti-*C. acnes* agents during acne treatment^44^. Since all strains were substantial biofilm producers, we next evaluated the potential correlation with the increased antibiotic tolerance. To this end, the antimicrobial susceptibility profiles were assessed in microbial isolates growing in biofilms.

Carbapenems, amoxicillin/clavulanic acid, piperacillin/tazobactam, and vancomycin were the most effective antibiotics against *C. acnes* biofilms with an MBEC comparable to the MIC values. However, biofilm-growing *C. acnes* exhibited a significant increase in the tolerance for ampicillin (*P* = 0.003), benzylpenicillin (*P* < 0.001), clindamycin (*P* < 0.001), and doxycycline (*P* < 0.001). Thus, the MBEC/MIC ratio, which indicates the fold increase in the antimicrobial dose needed to inhibit or kill *C. acnes* cells in biofilms compared to planktonic growth, was used to quantify the biofilm tolerance (BT) score (Fig. 6b). The maximum BT values were 32.1 and 17.6 reported for clindamycin, and the median BT values for the tested antimicrobials were between 2 (benzylpenicillin) and 4 (clindamycin and doxycycline).

## Discussion

*Cutibacterium* is one of the most abundant genera in the skin, particularly in sebum reach areas^50^, and its proliferation has been largely associated with acne^51,52^. Although, the specific role in the pathophysiology of acne vulgaris remains uncertain. To account for the complexity of the skin microbial environment, we analyzed the microbiota in different skin districts of healthy and acne patients. Our study demonstrated statistically significant differences in alpha and beta diversity between the skin of the healthy subjects and inflammatory lesions of acne patients. Specifically, mean alpha diversity was significantly reduced in LA compared with HS. This data suggests that the anaerobic and lipid-rich conditions within the pilosebaceous unit of inflammatory acne lesions may provide an optimal microenvironment for *C. acnes* growth, thus limiting potential competitors^53,54^. Previous metagenomic studies revealed that the relative abundances of *C. acnes* do not differ significantly between acne and healthy subjects^55–57^. However, the difference in *C. acnes* distribution could be ascribed to several variables, including the sequencing procedures and sampling methods^56^. The sequencing strategy and primer selection for 16S rRNA gene analysis are critical factors in characterizing the compositions of bacterial skin communities^58^. For example, the V1–V3 regions of the 16S RNA gene had greater accuracy than the V4 region in defining genus and species level classification of major skin bacteria^59^. In particular, V4 sequencing resulted in an inaccurate assessment of prominent skin bacteria showing lower relative abundances of *S. epidermidis* and *C. acnes* and higher relative abundance of *S. aureus* compared with V1–V3 sequencing^59^. Based on these data, we selected the V1-V3 region, which allowed us to effectively recover *Cutibacterium* and *Staphylococcus* species defining unbiased skin microbial community profiles and identifying potential microbial biomarkers associated with acne. The site and sampling technique can also affect the outcome of the sequencing-based analysis of the skin microbiome^60^. Previous studies reported that a swab is a reliable technique to analyze the skin microbiome comparable to the tape-stripping method^61,62^. In our study, microbiome samples were collected by the swab technique directly on LA and NI sites for each patient and compared with the same area of HS, providing a narrowed characterization of the local microbial population. The possibility of collecting microbiome samples from the lesional skin may have yielded more targeted results compared to other studies applying different sampling techniques. Indeed, others collected samples from the nose microcomedones of acne patients using adhesive strips. This sampling technique is ideal for identifying a homogeneous microbial population; however, it may not specifically reflect the microbial distribution present in the different microenvironments of acne^55^. In our study, the *Cutabacterium* genus was more abundant in LA and NI compared to HS. Accordingly, a significant increase in *C. acnes* was observed in LA compared to NI and HS. These results suggest that, although highly prevalent in the skin of HS and patients with acne, the relative abundance of *C. acnes* significantly increases in inflamed LA compared to NI. Therefore, the overgrowth of *C. acnes* in LA can potentially explain the prevalence of the disease in a part of the population, despite the universal carriage of the microorganism. These observations support previous findings suggesting that pilosebaceous unit obstruction and increasing *C. acnes* proliferation are likely central factors in the onset of inflammatory acne lesions^63,64^. This is consistent with the increase of *C. acnes* counts found in acne, which, in turn, correlates with the acne flares in adolescents, with high levels of hormones and sebum production^65^. Indeed, teenagers with acne have significantly higher *C. acnes* counts than age-matched controls, demonstrating a marked increase in this microorganism, promoting inflammation through various mechanisms^64,66,67^. Specific host factors, such as sebum production, hormone level, the inflammatory milieu, and physical changes in the pilosebaceous unit, may contribute to acne pathogenesis, indicating that certain strains can become pathogenic in different conditions or under specific environmental stimuli^16^. It has been suggested that inflammatory acne results from an imbalance in the skin microbiota associated with specific *C. acnes* phylotypes^35,68^. Our study found that the clonal complexes CC1 and CC3 were more abundant in LA than in HS; instead, CC4, CC5, and CC6 were present only in HS. In addition, IA1 was significantly more represented in LA than in HS, and the phylotypes IB and II were found only in the skin of HS. Data from this study are consistent with previous reports revealing that severe inflammatory acne lesions of both the face and back are associated with loss of diversity in *C. acnes* phylotypes, with a high predominance of phylotype IA1 compared to healthy controls^38,69,70^. Fitz-Gibbon et al., analyzed the distribution of *C. acnes* ribotypes (RT1, RT2, RT3) among both acne and normal follicles^55^, reporting that CC3 and CC4 of the phylotype IA1 were significantly enriched in patients with acne but rarely found in individuals with healthy skin. In contrast, RT6, which represents a subpopulation of phylotype II, was 99% associated with healthy skin. Interestingly, the non-acne-associated IB, types II and III, are also commonly recovered from deep tissue infections and retrieved from medical devices^16^. The loss of diversity between the six phylotypes, characterized by a dominance of IA1 rather than *C. acnes* proliferation, may play a key role in triggering acne^69–73^. As *C. acnes* is the major skin colonizer, the strain-level analysis is important to help understand the role of this bacterium in acne pathogenesis and skin health. Full genome sequencing of different strains from variable environmental sources, such as acne vulgaris and implant-associated infection, has revealed the pan-genome and the genetic repertoire of *C. acnes*^31,74–76^. Moreover, metagenomic analyses showed that several distinct virulence-associated gene elements that encode antimicrobial peptides, cytotoxins, and proteases were enriched in *C. acnes* strains associated with acne^12^. The accessory genome of *C. acnes* is relatively small, and non-core genes frequently code for a variety of phylotype-specific functions^31,74,75^. The variability at the phylotype level may correlate with the commensal or pathogenic phenotype of *C. acnes* and its contribution to acne^69^. In our study, the WGS analyses showed the CAMP factors were detected across all strains. Moreover, in accordance with previous observations, we reported that major virulence determinants are widespread among *C. acnes* and not specifically associated with any site of isolation or the phylotype^77^. Likewise, we observed that *luxS* and *roxP,* essential for adherence to and colonization of the skin, were ubiquitous in every *C. acnes* strain and not associated with specific genotypes. The high distribution of virulence genes across *C. acnes* isolates was previously described suggesting that transcription regulation may be critical in the differential virulence expression among phylotypes^78–80^. Nevertheless, the functional group analysis showed that the phylotype IA1 was characterized by a reduced number of carbohydrate metabolism and sugar transporters genes compared with phylotypes IB/II. This data suggests that carbohydrate transport and metabolism may confer to *C. acnes* an ecological advantage in healthy skin but are not strictly required for the selective colonization of inflammatory acne lesions. Indeed, previous studies hypothesized a connection between high sebum levels and *C. acnes* colonization in acne patients^81^. Accordingly, we observed that lipid transport and metabolism genes are increased in phylotype IA1 strains, compared with phylotypes IB/II, independently from the isolation site. Indeed, it is unlikely that phylotype IA1 strains isolated from LA have, per se, different properties than the same phylotype strains from healthy skin. In agreement with previous publications, we found no differences in the genomes and biofilm production of phylotype IA1 strains isolated from acne and healthy skin^82^. Future studies are required to define how virulence genes are expressed in different phylotypes. Previous imaging analysis showed that microcomedones' structure in acne patients resembles a pouch containing lipids with clusters of bacteria, whose outer shell comprises corneocyte layers^83^. The extensive bacteria colonization is visible using TEM^83^. The metabolism of *C. acnes* modifies the skin's lipid composition, and bacterial lipases release free fatty acids from triglycerides. Free fatty acids being more viscous than triglycerides, can obstruct the pilosebaceous unit, which in turn becomes anaerobic, promoting the selective proliferation of phylotype IA1 that better exploits the lipid-rich microenvironments of the pilosebaceous unit during acne compared to other phylotypes^83–85^. The analyses of the unique genes present in different phylotypes allowed us to identify that of 2769 genes, five genes with known functions were univocally present on all IA1 isolates (*acs*A, *clp*S, *dpp*B, *rcs*B, and *ytp*A). Notably, the *ytpA* gene, which encodes for a lysophospholipase that contributes to host-tissue degradation and inflammation, was present in all IA1 isolates.

To overcome these difficulties, a better understanding of the pathogenic potential of the individual sub-populations is needed. Additionally, others speculated that the persistent nature of acne vulgaris is linked to *C. acnes*’s colonization of the pilosebaceous unit in a biofilm, thereby eluding antibiotic eradication^40,86^. This theory was supported by observing that the genome of IA1 isolates contains genes such as *rcsB, acsA* that have a positive role in biofilm formation in other bacterial species^41,87–89^. Specifically, transcriptomic analysis of *Helicobacter pylori* revealed that several acetone metabolism genes, including *acsA*, are upregulated in biofilm cells^89^. The RcsCDB phosphorelay pathway coordinates the expressions of a large number of genes essential for maintaining cell wall integrity, cell division, stationary-phase sigma factor activity, biofilm development, motility, and virulence in Enterobacteriaceae^90,91^. RcsB upregulates genes promoting biofilm formation while downregulating different metabolic functions in *Escherichia coli*^87,88^. The comparative analysis of in vitro biofilm formation showed that the phylotype IA1 was significantly faster in early biofilm formation than IB and II strains in inhibiting the magnetic microparticles.

Moreover, biofilm quantification showed that IA1 strains produced significantly more mature biofilm than the other phylotypes confirming that both DNA and proteins are required for the early adhesion and biofilm formation in *C. acnes*^39^. In addition, our findings suggest that independently from the site of isolation, all the strains produce biofilm; however, phylotype IA1, which is the most prevalent in AP, resulted in the most effective. This notion is indirectly reinforced by confocal microscopy results, which clearly show the development of a thick and structurally complex biofilm matrix in the phylotype IA1. Furthermore, DNase I- and Proteinase K-sensitivity assays revealed that eDNA and proteins are central for early surface adhesion to abiotic surfaces, with eDNA playing a major role. Besides, an increased DNase I-sensitivity of phylotypes IB/II could be observed. Thus, in acne patients, a hyperseborrheic microenvironment, characterized by high availability of sebum, may result in an increased proportion of metabolically active biofilm-producing strains, providing a selective advantage to a subset of acne-associated phylotypes or sub-types, thus contributing to the proinflammatory milieu of acne lesions^54,65^.

Although antibiotics can reduce or eliminate *C. acnes*, recolonization frequently occurs within a few weeks, with limited clinical efficacy^44^. The high tolerance of *C. acnes* to antimicrobial treatments does not depend on the expression of multidrug-resistant genes since *C. acnes* are generally susceptible to most antimicrobials^44,92^. Conversely, the *C. acnes* capability of chronic persistence and relapse following antibiotic therapy is strongly suggestive of biofilm-related colonization^9,93^. These findings align with our results showing that all *C. acnes* isolates were highly susceptible to most antibiotics, with MIC values largely below the clinical breakpoints. However, the susceptibility profile of biofilm-growing *C. acnes* strains indicated a significant increase in antimicrobial tolerance against ampicillin, benzylpenicillin clindamycin, and doxycycline. For systemic treatment, oral tetracyclines (doxycycline, minocycline, oxytetracycline, or tetracycline) are the most recommended antibiotics for treating severe acne, followed by clindamycin due to relevant adverse effects^9^; while, topical antibiotic treatments mainly rely on clindamycin, erythromycin, and tetracycline^9^. These observations may explain the high tolerance of *C. acnes* to antimicrobial treatment and the conflicting evidence about the clinical benefit of using anti-*C. acnes* agents, even in cases where therapy was based on MIC results^44^. Besides, we found that vancomycin, piperacillin/tazobactam, and amoxicillin/clavulanic acid could effectively eradicate mature *C. acnes* biofilms, with BMIC similar to MIC. The most common and effective antibiotic against *C. acnes* in patients with prosthetic valve endocarditis was vancomycin^94,95^. In addition, we found that *C. acnes* isolates were highly susceptible to carbapenems in both planktonic and biofilm states. Previous research showed that carbapenems were highly active in vitro and in vivo against *C. acnes* strains isolated from postsurgical endophthalmitis^96^.

Our study suffers from some limitations. Indeed, the analysis herein was performed on a small group of patients. In addition, the in vitro condition for biofilm testing may not be fully representative of the real in vivo conditions of the pilosebaceous unit of acne patients. Moreover, only a single *C. acnes* isolate was selected from each patient. This procedure may not have captured the entire *C. acnes* phylotypes diversity in the facial region. However, a recent publication demonstrates that *C. acnes* colonies from the skin of adult subjects without acne vulgaris are often very closely related, suggesting the presence of person-specific populations^97^. Thus, further research will be required to elucidate whether these observations can be transferred in vivo*,* and any clinical findings should be interpreted with caution.

Overall, the results presented in this study indicate that the inflammatory lesions of acne patients are characterized by dysbiosis and decreased bacterial diversity. At the species level, *C. acnes* is significantly more abundant in LA than in NI and HS. Significant genotypic and phenotypic differences among *C. acnes* isolates were primarily dictated by the phylotype rather than the anatomical site of isolation. In particular, IA1 isolates were more efficient in early adhesion, biomass production, and antibiotic tolerance than other phylotypes. Thus, we hypothesize that in adolescent acne, the biochemical and physical changes of the pilosebaceous unit may promote dysbiosis, thus providing a selective advantage to a subset of metabolically active biofilm-producing phylotypes, such as IA1, that have the potential and the virulence, to outcompete commensals exacerbating the inflammatory response^98^. Accordingly, adolescent acne pathogenesis may not be caused by the mere presence of a disease-associated phylotype but rather by the response of the overall microbial community to microenvironmental changes of the pilosebaceous unit. Factors contributing to *C. acnes* growth compared to competitors during the progression from the non-inflamed to the inflamed acne lesions remain to be elucidated. Nevertheless, new antimicrobial agents targeting *C. acnes* biofilm matrix components may represent potential treatments to modulate the skin microbiota in adolescent acne.

## Methods

### Study design and patients’ enrolment

Patients with severe acne lesions were recruited among subjects at their first access to the Acne Unit at the San Gallicano Dermatological Institute. The control group was enrolled among HS undergoing mole checks in the same Institution. Informed consent was obtained from all the participants and their legal representative. Two dermatologists assessed the acne severity scores and the clinical grading. The intensity of clinical manifestations was evaluated on the face areas according to the Global European Acne severity (GEAS) criteria^99^. Briefly, subjects were classified as non-affected (clear) when no or very few lesions were clinically observable (0 ≤ GEAS < 1). GEA scores between 1 and 2 were referred to as the mild group, GEA scores 3 as the moderate group, and patients with GEAS 4–5 were classified as severe groups. Participants were asked to maintain their skincare routines except for prohibiting the use of cosmetics and moisturizing skincare products on the day of the enrolment. Criteria of inclusion were the absence of systemic diseases and skin disorders other than acne. The exclusion criteria were oral or topic pharmacological treatments at the first visit or up to 2 weeks before the examination, smoking, and use of sunbeds. The study was conducted according to the Helsinki declaration, and all enrolled patients signed informed consent. The Central Ethics Committee I.R.C.C.S. Lazio, the section of the Istituti Fisioterapici Ospitalieri in Rome, approved this study (Protocol 7679—21.06.2016, trials registry number 821/16).

### Sample collection

The samples were collected by dermatologists with commercially available sterile swabs (COPAN swabs, Brescia, Italy) from the skin of 10 HS and the unaffected skin and pustule of 10 acne patients in duplicate to assess the presence of C*. acnes* and for microbiome analysis. Swabs were brought to the Microbiology and Virology Laboratory of San Gallicano Institute for culture analysis and processed immediately in an anaerobic atmosphere. Schaedler Agar plates with 5% Sheep Blood (bioMérieux) were used to isolate and cultivate *C. acnes*. Plates were monitored for bacterial growth after 5–7 days of anaerobic incubation at 37 °C. Up to seven representative colonies of *C. acnes* were preliminarily identified by colony morphology and Gram staining. Further identification of *C. acnes* isolates was performed by MALDI-TOF mass spectrometry. Positive isolates were subcultured on Schaedler agar plates to obtain a pure culture of *C. acnes*. From each culture, bacterial DNA was obtained, and sequence analysis (ABI PRISM 3130xl Genetic Analyzer) of the 16SrRNA gene was used to confirm bacterial identification^100^. At the same time, the phylotype was preliminarily identified by Touch-down PCR^101^. Each sample's most common phylotype (detected in 85–100% of the colonies) was considered the dominant type. A single representative isolate of each dominant phylotype was selected for further analysis. *C. acnes* isolates were frozen and stored at − 80 °C. The co-hemolytic reaction of the CAMP factor on blood agar plates (bioMérieux) was performed using *S. aureus* strain ATCC 25923, according to the previous method^23^.

### Sequencing and analysis

Extracted DNA was amplified by PCR with dual-index primers targeting the V1-V3 regions of the bacterial 16S rRNA gene, using the ARROW for NGS Microbiota solution A kit (ARROW Diagnostics) according to the manufacturer’s instruction. A sterile sample tube that had undergone the same DNA extraction and PCR amplification procedures was used for quality control^102^. Before sequencing, amplicons were purified using the Agencourt® AMPure XP PCR purification system (Beckman Coulter, Milan, Italy), and equal amounts (10 nM) of the sample’s DNA were pooled and diluted to reach a 4-nM concentration. Finally, a 5 pM of the denatured library was used to generate sequences using the 2 × 250 cycles MiSeq Reagent kit (Illumina) on an Illumina MiSeq instrument. Sequencing data were analyzed using the MicrobAT system^102,103^. During MicrobAT processing, demultiplexed sequences showing reads of length less than 200 nucleotides, an average Phred quality score below 25, and at least one ambiguous base were discarded^104^. The resulting sequences were aligned at a 97% sequence similarity and assigned to taxonomic (e.g., species) levels at an 80% classification threshold using the Ribosomal Database Project (RDP) classifier (release 11.5)^105^. Species that did not meet these criteria were assigned to the corresponding group, “unclassified [genus]”. The Biological Observation Matrix (BIOM) was obtained, and the following analysis was carried out in R studio (https://www.rstudio.com/; version 4.0.2) using the phyloseq package^106^. Microbial community differences were measured in terms of alpha and beta diversity after reading depth rarefaction. Shannon index and Pielou index were used to evaluate alpha diversity, and significance was assessed by the Kruskal Wallis test. Bray Curtis beta diversity was calculated, and the distance matrix was represented as Principal coordinate analysis (PCoA)^107^. Significance was assessed by Permutational multivariate analysis of variance (PERMANOVA)^108^. Bacterial relative abundances at phylum and genus level between selected groups were examined.

### Cutibacterium* acnes* typing

FASTQ files, including Quality Control, trimming, assembly, and gene annotation, were elaborated using the Bactopia suite^109^. The pan-genome was obtained using Roary^110^ on the annotated assemblies. Functional annotation was performed using EggNOG v5.0^111^ on the protein sequences. The Phylotypes and Clonal Complexes were determined by querying the API of PubMLST (www.pubmlst.org) using custom Python scripts. Whole-genome analysis of variants (SNPs/indels) on the IA1 strains and the phylogenomic tree was obtained as described previously^112^. The independent analysis of the core genome was performed using PIRATE^113^ on the IA1 strains to build a phylogenomic tree.

### Biofilm Ring Test® (BRT)

Biofilm production was evaluated using the clinical BioFilm Ring Test (cBRT) with some modifications^114^. Briefly, overnight bacterial cultures grown on Schaedler agar plates + 5% sheep blood (bioMérieux, France) was used to inoculate 2 mL of 0.45% saline solution (AirLife, Carefusion, CA, USA) to the equivalent of 2.5 ± 0.3 McFarland turbidity standard. The bacterial suspension was used to inoculate a 96-well polystyrene plate with 200 μL/well. The test was performed using toner solution (TON) (Biofilm Control, Saint Beauzire, France) containing magnetic beads 1% (v/v) mixed in Brain Heart Infusion medium (BHI, Difco, Detroit, MI, USA). Sample dilutions (tenfold serial dilutions, from 1 × 10^–1^ to 1 × 10^–3^) were performed in a volume of 200 μL BHI/TON mix. The laboratory strain *C. acnes* ATCC 11827 was included in each test as standard reference and quality control. A well containing the BHI/TON mix without microbial cells was used in each experiment as a negative control. Plates were incubated at 37 °C without shaking (static culture) under anaerobic conditions. Biofilm formation was assessed at different time points. After incubation, wells were covered with few drops of contrast liquid (inert opaque oil used), placed for 1 min on the block carrying 96 mini-magnets (Block test), and scanned with a specifically designed plate reader (Pack BIOFILM, Biofilm Control, Saint Beauzire, France)^115^. Each strain was analyzed in duplicate, and experiments were repeated three times.

### Assessment of *C. acnes* biofilm composition

The Biofilm Ring Test method was used to evaluate the early attachment in the presence of DNase I (100 μg/mL) and proteinase K (50 μg/mL)^49,116^. Standardized bacterial suspensions containing 1 vol % magnetic beads were supplemented with DNase (100 μg/mL) and proteinase K (50 μg/mL) and incubated at 37 °C in a 96-well microplate (200 μL/well). Negative controls contained 200 μL of sterile BHI with magnetic beads and enzymes. The plate was read after 6 h of incubation, as described above. The early adhesion in the presence of DNase and proteinase K was expressed using the relative difference (RD):

1 $$RD = \left[ {\left( {{\text{Pmb}}\,{\text{without}}\,{\text{enzyme}}{-}{\text{Pmb}}\,{\text{with}}\,{\text{enzyme}}} \right)/{\text{Pmb}}\,{\text{without}}\,{\text{enzyme}}} \right] \times 100$$

The analysis was performed three times in duplicate for each sample.

### Evaluation of the biofilm formation with the crystal violet staining

Sterile 96-well polystyrene plates were inoculated with 200 μL of an initial bacterial suspension (10^5^ CFU/mL) in BHI broth incubated at 37 °C for 72 h in anaerobic conditions without shaking. As described previously, biofilm formation was assayed using crystal violet staining in 96-well microtitre plates^114^.

### Susceptibility testing

#### Minimum biofilm inhibitory concentration (MIC)

MICs were determined for each strain using the broth microdilution method described previously^116^ and adapted to the specific growth conditions of *C. acnes*. Specifically, *C. acnes* strains grown on Schaedler agar plates were inoculated into 2 mL of 0.45% saline solution (Air Life, Carefusion, CA, USA) to obtain turbidity of 0.5 ± 0.1 McFarland turbidity standard (approximately 10^8^ CFU/mL). Samples were diluted at 1:100 in BHI, and 100 μL of bacterial suspension, were seeded into a sterile 96-multiwell polystyrene plate containing different antibiotics at variable concentrations (Corning Inc., Corning, NY, USA). Serial two-fold dilutions of the amoxicillin/clavulanic acid, ampicillin, benzylpenicillin, clindamycin, doxycycline, ertapenem, imipenem, meropenem, moxifloxacin, piperacillin/tazobactam, tigecycline, vancomycin were prepared. After the antibiotic treatment, viable cells were determined by plate counting for the CFU/mL determination. The MIC was defined as the lowest concentration of an antibiotic preventing bacterial growth. Experiments were conducted in triplicate.

#### Minimal biofilm eradication concentration (MBEC) assays

For each experiment, an overnight culture of *C. acnes* grown on a blood agar plate was used to inoculate 2 mL of 0.45% saline solution to 0.5 ± 0.1 McFarland turbidity standard. For biofilm cultures, diluted cell suspensions (approximately 10^6^ CFU/mL) were used to inoculate a 96-well polystyrene flat-bottom plate with 100 mL BHI. After 24 h at 37 °C, in anaerobic conditions, the wells were rinsed with 0.45% saline solution to remove nonadherent bacteria. The plate was washed three times with distilled water, and the adherent cells were resuspended in 100 mL of BHI supplemented with two-fold serial dilutions of amoxicillin/clavulanic acid, ampicillin, benzylpenicillin, clindamycin, doxycycline, ertapenem, imipenem, meropenem, moxifloxacin, piperacillin/tazobactam, tigecycline, vancomycin. After overnight treatment, antibiotics were removed, and the plate was washed twice with 200 μL of sterile distilled water. Biofilms were scraped thoroughly, and the total number of viable cells was determined by serial dilution and plating on Schaedler agar plates to estimate the CFU number. The MBEC was defined as the lowest concentration of an antibiotic agent preventing bacterial growth.

MBEC/MIC-ratios were calculated to assess the biofilm tolerance (BT) score, which indicates the fold increase in the antimicrobial dose needed to inhibit or kill *C. acnes* cells in biofilm compared to planktonic growth^116^.

### Biofilm Imaging

*Cutibacterium acnes* colonies, grown overnight on Schaedler agar plates, were used to inoculate 3 mL of 0.45% saline solution (Air Life, Carefusion, CA, USA) to obtain turbidity of 2.5 ± 0.3 McFarland turbidity standard corresponding approximately to 1 × 10^8^ CFU/mL. Samples were diluted 1:1000 and resuspended in 1 mL of BHI in a μ-Slide, 8 well glass bottom chamber slides (Ibidi, Germany). The bacterial suspension was incubated at 37 °C for 72 h to allow biofilm formation. Subsequently, the medium was removed, and samples were washed in a 0.45% saline solution. According to supplier specifications, the biofilm cells were stained using the LIVE/DEAD BacLight kit (Life Technologies, New York, NY, USA)^116^ and examined with a Zeiss LSM5 Pascal Laser Scan Microscope (Zeiss, Oberkochen, Germany) Software Release 2.8 (Zeiss).

### Statistics

Data were expressed as the mean ± standard error of the mean. Statistical analysis was performed using either ANOVA with Tukey post-hoc or Kruskal–Wallis with Dunn’s post-hoc tests, followed by appropriate *p*-value correction. For Beta diversity, PERMANOVA was used on Bray–Curtis distance matrices. *P*-values of 0.05 or less were considered statistically significant. IBM SPSS v.21 statistics software (IBM, Chicago, IL, USA) was used for all statistical analyses.

## Supplementary Information

Supplementary Legends.

Supplementary Figure S1.

Supplementary Figure S2.

## Data Availability

The authors declare that the data supporting the findings of this study are available within the paper or from the corresponding author upon reasonable request. The data for this study have been deposited in the European Nucleotide Archive (ENA) at EMBL-EBI under accession number PRJEB55087 (https://www.ebi.ac.uk/ena/browser/view/ PRJEB55087).
