# Supplementary material for: Skin dysbiosis and Cutibacterium acnes biofilm in inflammatory acne lesions of adolescents
Source: Sci Rep. 2022 Dec 6;12:21104. doi: 10.1038/s41598-022-25436-3 (PMC9727105; doi:10.1038/s41598-022-25436-3)
Supplement: Supplementary file 1 — Supplementary Legends. [file 41598_2022_25436_MOESM1_ESM.docx]

**Supporting Information:**

This research was funded by the Italian Ministry of Health (RC 2022)

**Additional information**

**Supplementary Information**

Figure S1: Phylogenetic tree of the indicated strains based on the core pangenome, built using PIRATE. Additional *C. acnes* assemblies named "GCF {*number*}" were downloaded from RefSeq. ST: sequence type; CC: clonal complex; HS: healthy skin; LA: lesional acne.

Figure S2: Phylogenomic tree based on the strains core variants alignment, built using Snippy. ST: sequence type; CC: clonal complex; HS: healthy skin; LA: lesional acne.
