# Supplementary figures and images for: Skin dysbiosis and Cutibacterium acnes biofilm in inflammatory acne lesions of adolescents
Source: Sci Rep. 2022 Dec 6;12:21104. doi: 10.1038/s41598-022-25436-3 (PMC9727105; doi:10.1038/s41598-022-25436-3)

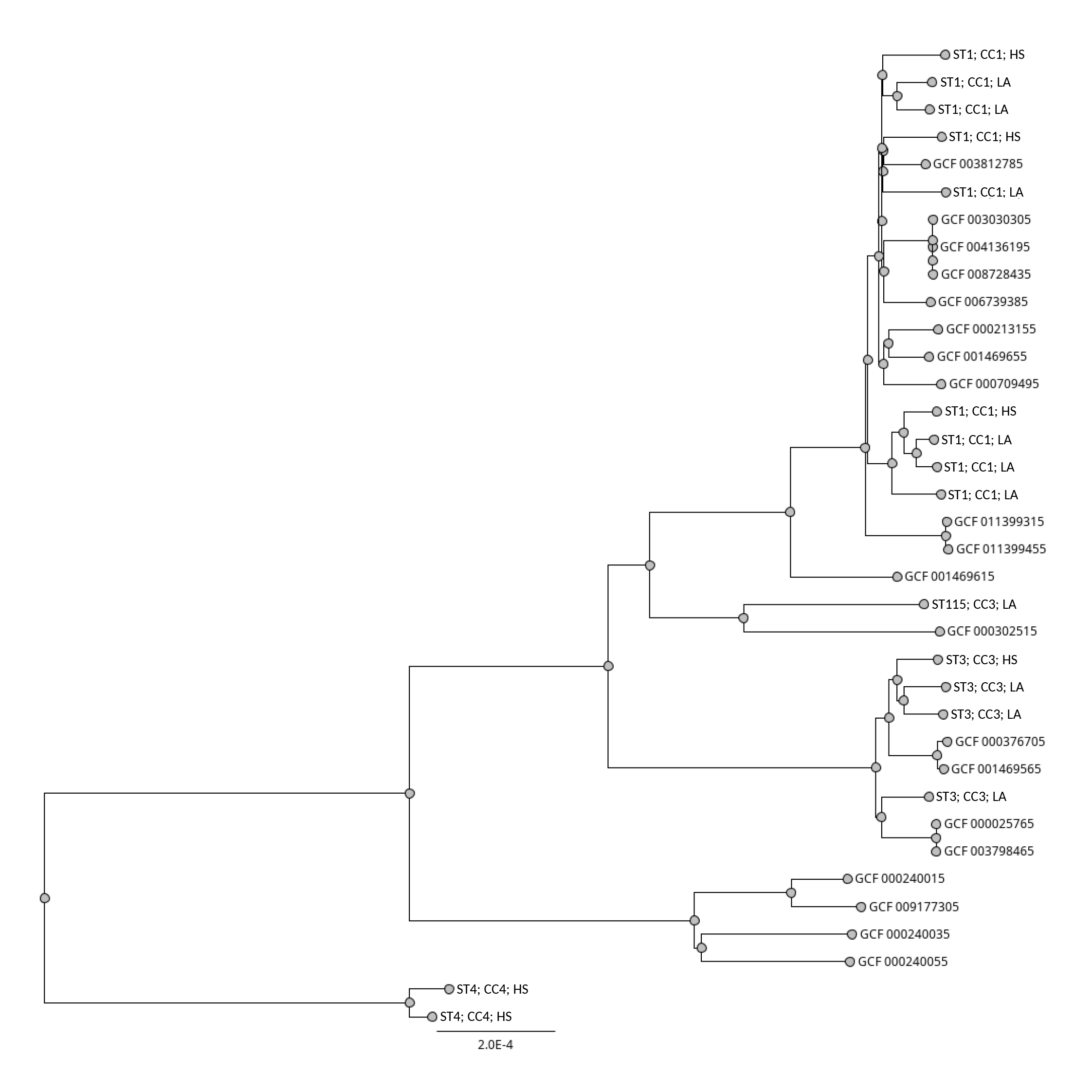

Supplement: Supplementary file 2 — Supplementary Figure S1. [file 41598_2022_25436_MOESM2_ESM.tif]

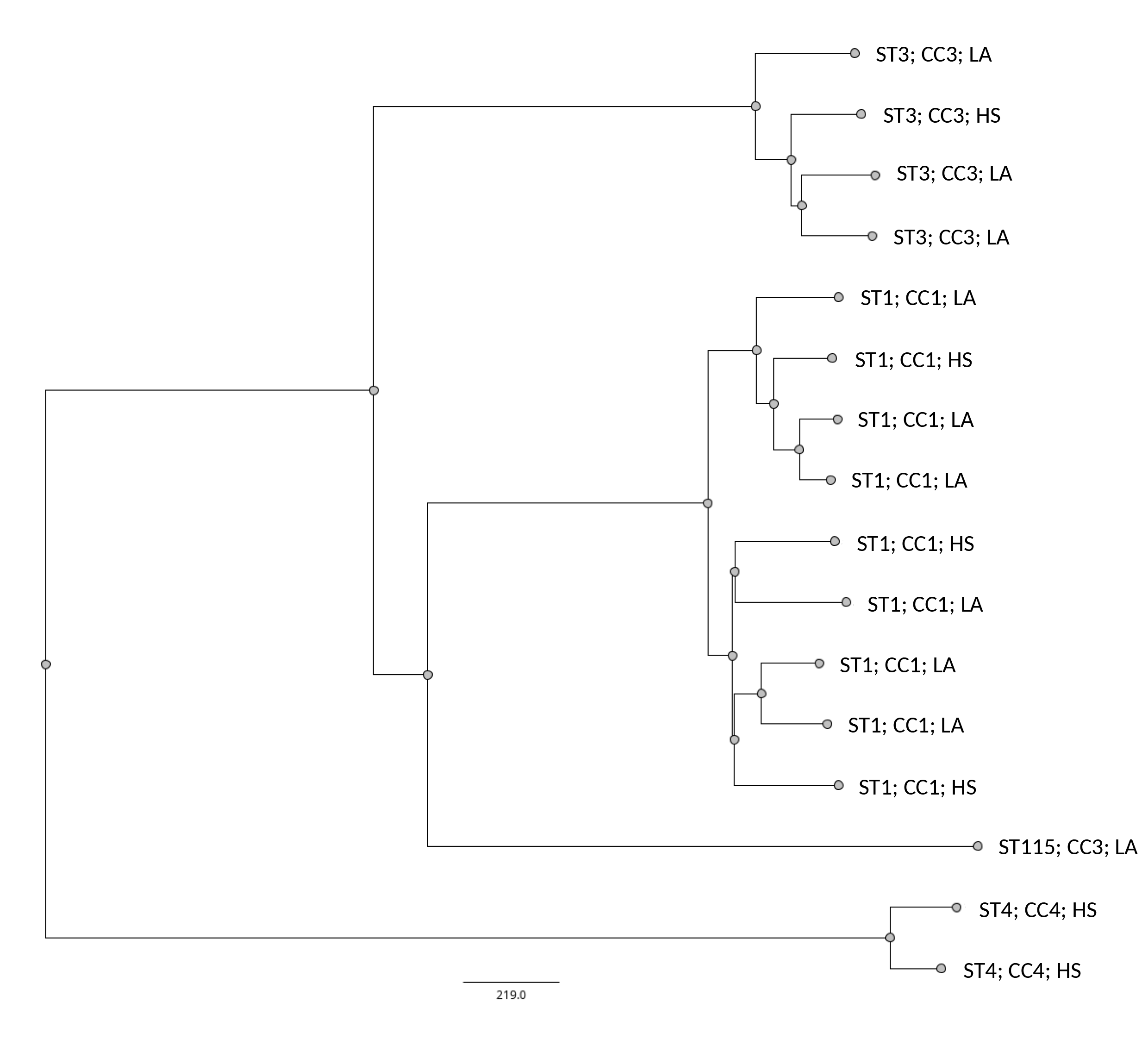

Supplement: Supplementary file 3 — Supplementary Figure S2. [file 41598_2022_25436_MOESM3_ESM.tif]
